# Hepatotoxin-Induced Changes in the Adult Murine Liver Promote MYC-Induced Tumorigenesis

**DOI:** 10.1371/journal.pone.0002493

**Published:** 2008-06-18

**Authors:** Shelly Beer, Kimberly Komatsubara, David I. Bellovin, Masashi Kurobe, Karl Sylvester, Dean W. Felsher

**Affiliations:** 1 Department of Medicine, Division of Oncology, School of Medicine, Center for Clinical Sciences Research, Stanford University, Stanford, California, United States of America; 2 Department of Pathology, Division of Oncology, School of Medicine, Center for Clinical Sciences Research, Stanford University, Stanford, California, United States of America; 3 Department of Surgery, Division of Pediatric Surgery, School of Medicine, Stanford University, Stanford, California, United States of America; University of Helsinki, Finland

## Abstract

**Background:**

Overexpression of the human c-MYC (MYC) oncogene is one of the most frequently implicated events in the pathogenesis of hepatocellular carcinoma (HCC). Previously, we have shown in a conditional transgenic mouse model that MYC overexpression is restrained from inducing mitotic cellular division and tumorigenesis in the adult liver; whereas, in marked contrast, MYC induces robust proliferation associated with the very rapid onset of tumorigenesis in embryonic and neonatal mice.

**Methodology/Principal Findings:**

Here, we show that non-genotoxic hepatotoxins induce changes in the liver cellular context associated with increased cellular proliferation and enhanced tumorigenesis. Both 5-diethoxycarbonyl-1,4-dihydrocollidine (DDC) and carbon tetrachloride (CCl_4_) cooperate with MYC to greatly accelerate the onset of liver cancer in an adult host to less than 7 days versus a mean latency of onset of over 35 weeks for MYC alone. These hepatotoxin-enhanced liver tumors grossly and histologically resemble embryonic and neonatal liver tumors. Importantly, we found that MYC overexpression is only capable of inducing expression of the mitotic Cyclin B1 in embryonic/neonatal hosts or adult hosts that were treated with either carcinogen.

**Conclusion/Significance:**

Our results suggest a model whereby oncogenes can remain latently activated, but exposure of the adult liver to hepatotoxins that promote hepatocyte proliferation can rapidly uncover their malignant potential.

## Introduction

MYC is a proto-oncogene that regulates normal cellular growth, proliferation, apoptosis, and differentiation [1]. MYC is frequently overexpressed or mutated in human cancers and is thought to contribute to tumorigenesis by inducing autonomous cellular growth and proliferation, blocking differentiation, and inducing genomic instability [1]–[5]. Aberrant MYC oncogene expression has been frequently observed in primary human and rodent liver tumors [6]–[8]. Importantly, MYC expression is sufficient to induce tumor formation in murine models, with liver-specific overexpression directed by the albumin enhancer/promoter or the alpha-1-antitrypsin promoter resulting in HCC [9], [10]. However, the tumor incidence in these mice is relatively low and the latency is long. Notably, when MYC is co-expressed in the murine liver with either Transforming growth factor alpha (TGF-alpha) or E2F transcription factor 1 (E2F1), the onset of tumorigenesis is significantly accelerated [11]–[13]. These data suggest that MYC activation can be significantly augmented by various complementary stimuli, providing a system by which modifiers of the tumor phenotype may be revealed.

**Figure 1:**
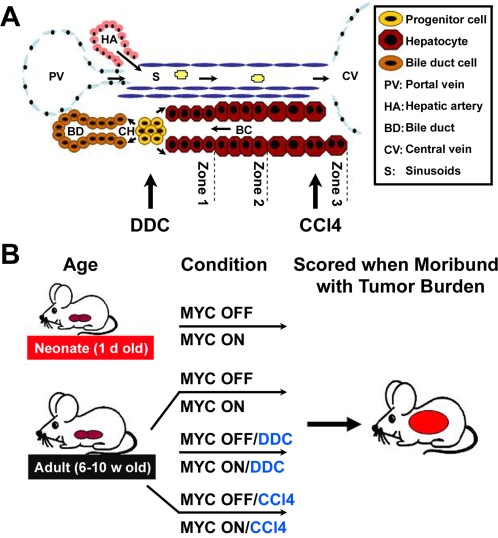
Examining the Impact of DDC and CCl_4_ Damage on MYC-Induced HCC. (A) Schematic of the liver lobule illustrating that DDC causes liver damage associated with the emergence and proliferation of oval cells in the periportal area of the hepatic lobule, while CCl_4_ causes damage associated with the destruction of hepatocytes near the central vein and triggers mature hepatocytes to proliferate. (B) Schematic of experimental design. Eight different cohorts of mice were monitored for tumorigenesis. MYC was activated (MYC ON) or kept inactive (MYC OFF) in mice: at birth, in 6–10 week old adult, in 6–10 w old adult mice treated with DDC, or in 6–10 w old adult mice treated with CCl_4_. Mice were sacrificed when moribund with tumor burden.

Epidemiological studies in humans, as well as data from animal models, support the idea that the liver is more susceptible to neoplastic transformation during states of liver growth and regeneration [14], 15. Patients with alcohol-induced cirrhosis of the liver were found to be at greater risk of developing hepatocellular carcinoma than those without cirrhosis [16]. Similarly, various hepatotoxins have been reported to enhance liver tumorigenesis in many contexts, including mice that are transgenic for hepatitis b virus (HBV) protein or TGF-alpha overexpression [17]–[19]. Together, these data support the model of accelerated liver tumorigenesis as a result of changes in the liver. Notably, different hepatotoxins damage distinct regions of the liver lobule and have been shown to elicit different cells in the hepatic lineage to restore hepatocyte loss during tissue regeneration, thereby influencing the cellular origin of HCC (Figure 1A) [20], [21]. Based on the differential effect of various toxins, some chemical carcinogenesis models have favored the hepatocyte as the precursor to HCC, while others have implicated liver progenitor cells (oval cells) as the cell of origin for HCC [22]–[24].

Recently, we have described the use of the tetracycline-regulated (Tet) system to develop a conditional transgenic model of MYC-induced HCC [25], [26]. Using this system, we found that MYC's ability to induce mitotic division and tumorigenesis in the liver is developmentally regulated [26]. In the permissive context of an embryonic or neonatal liver, in which hepatocytes are actively undergoing mitotic cellular division, MYC overexpression further increased cellular proliferation and resulted in the almost immediate onset of liver tumorigenesis. In contrast, when overexpressed in mitotically quiescent adult murine hepatocytes, MYC was capable of inducing cellular growth and DNA replication, but was prohibited from inducing mitotic division and resulted in HCC only after a prolonged latency. Hence, the cellular and developmental context of the host appears to play a critical role in defining the ability of MYC to induce tumorigenesis in the liver.

Non-genotoxic hepatotoxins are examples of agents that could be responsible for changes in the liver that would enhance the ability of oncogenes such as MYC to induce liver tumorigenesis. CCl_4_ is a well characterized carcinogen that causes centrilobular destruction of hepatocytes and triggers mature hepatocytes to proliferate and reconstitute the lost liver mass (Figure 1A) [27], [28]. Other agents such as DDC have also been reported to cause liver damage associated with the proliferation of oval cells in the periportal area of the hepatic lobule, however, as of yet have not been reported to be carcinogens (Figure 1A) [29]–[31]. Here we show that both DDC and CCl_4_ similarly facilitate MYC-induced initiation of hepatocyte proliferation and tumorigenesis in the adult liver. Our findings suggest a model whereby external stimuli may unveil latent oncogene activation in the mature liver.

## Results

### DDC and CCl_4_ treatment accelerate MYC-induced HCC in adult hosts

**Figure 2:**
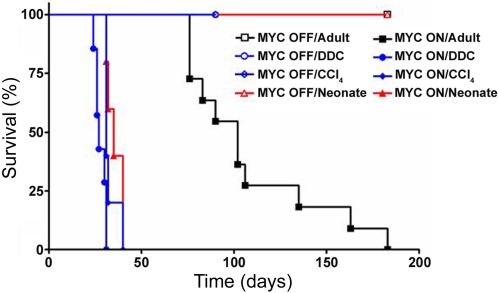
DDC and CCl_4_ Cooperate with MYC to Induce Accelerated Liver Tumorigenesis. Shown are Kaplan-Meier survival curves for 6–8 w adult mice that overexpressed MYC and were simultaneously treated with: nothing (filled black square), DDC (filled blue circle), or CCl_4_ (filled blue diamond). Adult mice treated with hepatotoxins in the absence of MYC overexpression (MYC OFF) are displayed with the corresponding open shape: nothing (empty black square), with DDC (empty blue circle), or CCl_4_ (empty blue diamond). Also shown are survival curves of neonatal mice in which MYC was either overexpressed (filled red triangle), or remained inactive (empty red triangle) starting at birth. Cohorts consisted of 5–10 mice. MYC transgene expression was activated by removal of doxycycline from the mouse drinking water on the day of hepatotoxin treatment initiation. Mice were sacrificed when moribund with tumor burden.

To examine the effects of DDC on MYC's ability to induce tumorigenesis in the adult liver, 6-week-old adult mice were fed a diet containing 0.1% DDC [29]. MYC was induced in one cohort of mice (MYC ON/DDC) and kept inactive in controls (MYC OFF/DDC) (Figure 1B). To distinguish between DDC-specific and other hepatotoxin effects, a separate cohort of transgenic mice were injected with CCl_4_ twice weekly for the duration of the experiment (4 months), either in the presence (MYC ON/CCl4) or absence (MYC OFF/CCl4) of MYC overexpression (Figure 1B). As an additional control, adult mice in which MYC was overexpressed by itself (MYC ON) or kept inactive (MYC OFF) were also monitored (Figure 1B). Remarkably, mice that overexpressed MYC in conjunction with either DDC or CCl_4_ treatment (MYC ON/DDC and MYC ON/CCl4) developed liver cancers at a substantially accelerated onset as compared to the control MYC ON mice (Figure 2). Specifically, the rate at which all MYC ON/DDC and MYC ON/CCl4 animals were moribund with tumor burden was 31 and 40 days, respectively, compared to 183 days for MYC ON by itself. Indeed, by the time hepatotoxin-associated mortality was at 100%, the MYC ON cohort had yet to demonstrate morbidity. Interestingly, the rapid onset of liver tumorigenesis in MYC ON/DDC and MYC ON/CCl4 mice was similar to that observed when MYC was overexpressed in neonatal livers (Figure 2). Mice in which MYC was inactive (MYC OFF/DDC, MYC OFF/CCl4 and MYC OFF) remained tumor free for the duration of the experiment as expected (4 months, Figure 2).

### DDC and CCl_4_ cooperate with MYC to induce diffuse HCC

**Figure 3:**
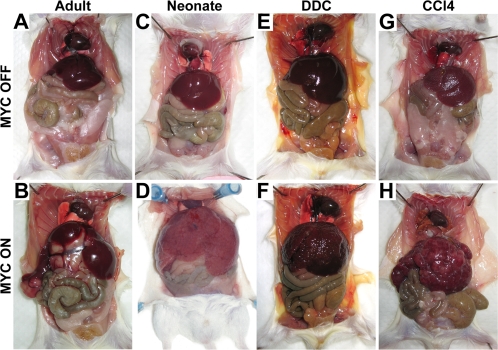
DDC and CCl_4_ Cooperate with MYC to Induce Diffuse HCC. (A) A normal adult liver (MYC OFF Adult). (B) An adult liver tumor (MYC ON Adult). (C) A normal 4 w old liver (MYC OFF Neonate). (D) A neonatal liver tumor (MYC ON Neonate). (E) An adult liver 3–4 weeks after continuous DDC treatment (MYC OFF/DDC). (F) An adult liver tumor induced by MYC overexpression in conjunction with continuous DDC treatment (MYC ON/DDC). (G) An adult liver 4 weeks after continuous CCl_4_ treatment (MYC OFF/CCl_4_). (H) An adult liver tumor induced by MYC overexpression in conjunction with continuous CCl_4_ treatment (MYC ON/CCl_4_). Representative animals from each cohort are presented. Note that toxin-accelerated HCC are diffuse, similar in appearance to neonatal tumors. In contrast, normal MYC-induced adult tumors present as a distinct, multifocal disease.

Since tumor latency was dramatically reduced upon hepatotoxin treatment, it was necessary to investigate whether the resulting disease appeared phenotypically similar. Upon gross examination, MYC ON/DDC and MYC ON/CCl4 mice developed tumors associated with three-fold enlarged livers with a multitude of coalescing tumor nodules (Figure 3F, H). As observed with tumor latency, hepatotoxin-accelerated HCC appeared similar to the MYC-induced neonatal livers, demonstrating a diffuse tumor phenotype (Figure 3D) [26]. In contrast, adult mice that overexpressed MYC by itself developed enlarged livers with multiple discrete tumor nodules, as previously described (Figure 3B) [26]. Although MYC OFF/DDC mice did not develop tumors, they exhibited progressive abdominal enlargement during the duration of DDC feeding, reaching a peak at about 4 weeks. Grossly, this abdominal growth was associated with 2-fold enlarged livers (Figure 3A, E). In contrast, MYC OFF/CCl4 livers were either equal to or 20% smaller than a normal liver and associated with a nodular appearance (Figure 3A, G).

### MYC expression does not enhance hepatotoxin-induced oval cell expansion

**Figure 4:**
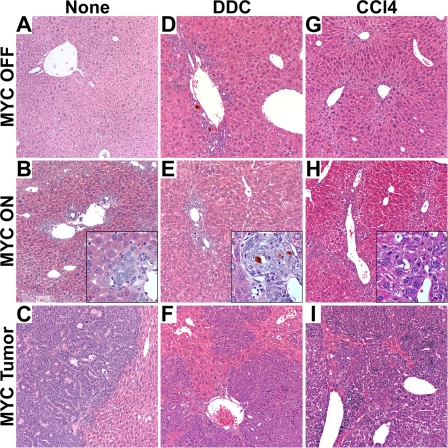
Hepatotoxins Induce Liver Toxicity and Cooperate with MYC to Induce Diffuse HCC's Arising from Periportal Areas. (A) Histology of a normal adult liver. (B) Histology of a MYC-induced microcarcinoma that developed in a periportal area of the liver. Inner panel is a magnified view of the microcarcinoma. (C) Histology of a MYC-induced, multifocal adult liver tumor. (D) Histology of an adult liver 3 w after continuous DDC treatment. The liver lobule exhibits oval cell expansion radiating from the periportal area, typical of DDC-induced injury. (E) Histology of a MYC and DDC-induced microcarcinoma that stems from the periportal area. Inner panel is a magnified view of the microcarcinoma. (F) Histology of a MYC and DDC-induced diffuse liver tumor. (G) Histology of an adult liver after 4 weeks of continuous CCl_4_ treatment. Microvesicular lipidosis can be seen in the centrilobular area of the liver lobule. (H) Histology of a MYC and CCl4-induced microcarcinoma that stems from the periportal area. Inner panel is a magnified view of the microcarcinoma. (I) Histology of a MYC and CCl_4_-induced diffuse liver tumor. Representative data is shown.

To further characterize the effects of DDC and CCl_4_ on MYC-induced HCC formation in adult liver, histological analysis was performed. In MYC OFF/DDC livers, we found evidence for liver injury associated with the expansion of oval cells near the periportal areas (Figure 4D). This oval cell expansion reached a peak three-to-four weeks after DDC diet initiation, consistent with previous findings [29], and may have contributed to the marked liver growth. MYC OFF/CCl4 mice developed liver injury associated with post-necrotic lobular collapse (Figure 4G). This hepatocyte death and necrosis may explain the decrease in size of MYC OFF/CCl4 livers relative to normal livers (Figure 3A, G).

In addition, MYC ON/DDC and MYC ON/CCl4 livers exhibited evidence for microcarcinomas as early as ten days after MYC activation (Figure 4E, H), prior to developing diffuse HCC (Figure 4F, I). Eighty-six percent of microcarcinomas found in MYC ON/DDC, and 50% of those found in MYC ON/CCl4 livers were located adjacent to periportal areas of the liver lobule (Figure 4E, H). MYC ON adult mice also developed 93% of microcarcinomas near the periportal areas, although after a much longer latency period (Figure 4B). These results are consistent with the previously suggested notion that many HCC's derive from the malignant expansion of less mature hepatocytes or oval cells [23].

**Figure 5:**
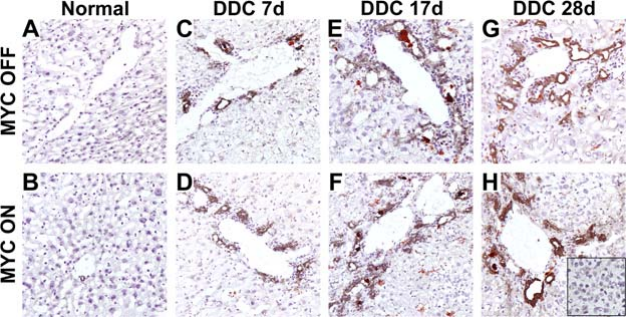
MYC Overexpression Does Not Contribute to Oval Cell Expansion. A6 Immunohistochemistry of: (A) a normal adult liver, (B) an adult liver in which MYC has been activated for 30 days, (C) an adult liver treated with DDC for 7 days, (D) an adult liver that overexpressed MYC in conjunction with DDC treatment for 7 days, (E) an adult liver treated with DDC for 17 days, (F) an adult liver that overexpressed MYC in conjunction with DDC treatment for 17 days, (G) an adult liver treated with DDC for 28 days, and (H) an adult liver with adjacent tumor foci that stemmed from MYC and DDC treatment for 28 days. A6 positive cells are evident in the periportal areas but not in the tumors. Representative data is shown.

In order to address whether the reduced tumor latency observed in DDC-treated mice may have been due to an increase in the oval cell population, immunohistochemistry (IHC) was employed. Accordingly, an antibody specific for the A6 antigen, known to be expressed on oval cells, was utilized [32]. Prior to induction, few cells were reactive for the A6 antigen, as expected (Figure 5A). Upon initiation of DDC treatment, oval cells emerged in the periportal areas by 7 days (Figure 5C) and continued to expand, reaching a peak between 3 and 4 weeks (Figure 5E, G). It was expected that, if oval cell expansion was critical for increased tumorigenesis, the induction of MYC overexpression together with DDC treatment would result in a significant increase in these cells. However, the increase observed in oval cells was comparable between MYC ON and MYC OFF conditions at all time points (Figure 5A–H), suggesting that MYC overexpression may not contribute to further oval cell expansion in the liver. Furthermore, no A6 positive cells were detected in MYC ON/DDC-induced neoplastic foci (Figure 5H) or in pre-neoplastic livers in which MYC was expressed for 30 days (Figure 5B). One possible explanation for these data is that some oval cells were undergoing neoplastic transformation, or, alternatively, differentiating into immature hepatocytes and thus were no longer expressing the A6 antigen.

### DDC and CCl_4_ facilitate MYC-induced mitotic cellular division and proliferation in the adult liver

**Figure 6:**
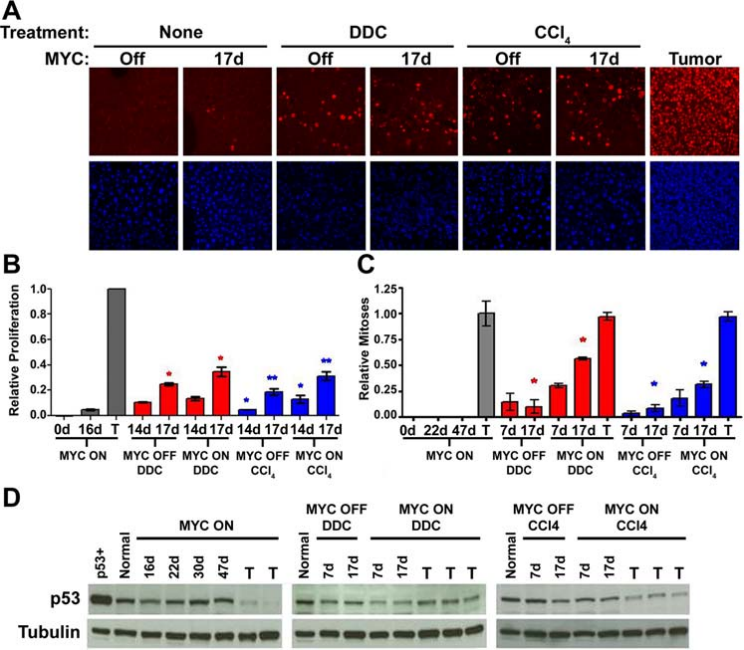
Hepatotoxins Facilitate MYC-Induced Proliferation Irrespective of p53 Status. (A) Immunofluorescence for Ki67 expression as a marker of liver cell proliferation is presented. (B) Bar graphs illustrating a ratio of Ki67 positive hepatocytes to total hepatocytes as a relative measure of hepatocyte proliferation are shown. (C) Bar graphs illustrate a ratio of phospho-Histone-H3 positive hepatocytes to total hepatocytes as a relative measure of hepatocyte mitotic division. Gray bar graphs represent normal adult livers in which MYC was either overexpressed (MYC ON/Adult), or kept inactive (MYC OFF/Adult). Red bar graphs represent DDC-treated livers in which MYC was either overexpressed (MYC ON/DDC), or kept inactive (MYC OFF/DDC). Blue bar graphs represent CCl_4_-treated livers in which MYC was either overexpressed (MYC ON/ CCl_4_), or kept inactive (MYC OFF/ CCl_4_). T represents a liver tumor. Results are based on an average of 4 samples per time-point. Statistical significance was measured using a Mann Whitney test: graph in (A) (* P<0.05, ** P<0.05), and graph in (B) (* P<0.05). (D) Western blot analysis for p53 protein expression after different durations of MYC overexpression in: Normal, DDC-treated, and CCl_4_-treated livers. As a positive control, a lymphoma cell line that overexpressed a mutant p53 was used. Normal FVB/N adult liver was used as a negative control.

In the adult murine liver, the ability of MYC to initiate tumorigenesis is associated with the ability to induce the cellular growth and DNA replication but not mitotic division of hepatocytes [26]. It has been demonstrated that continuous exposure to hepatotoxins such as DDC and CCl_4_ causes liver injury that is accompanied by tissue regeneration. We speculated that DDC and CCl_4_ were contributing to accelerated MYC-induced HCC by altering the liver context, thereby enhancing the mitotic division of hepatocytes. As such, liver cell proliferation was measured by Ki67 immunofluorescence and IHC for phosphorylated Histone H3 (Figure 6A–C). DDC and CCl_4_ treatments both resulted in a significant increase in the proliferative index, regardless of MYC expression. Indeed, either DDC or CCl_4_ treatment for 17 days alone was sufficient to increase cellular proliferation and mitosis above that resulting from MYC overexpression for the same duration. However, this effect was exaggerated when combined with oncogene activation. As measured by Ki67, MYC ON/DDC and MYC ON/CCl4 livers exhibited a 30 and 40 percent increase in hepatocyte proliferation, respectively, as compared to DDC and CCl4-treated MYC OFF livers (Figure 6A). In addition, a 300 and 250 percent increase in hepatocyte mitotic division as measured by phospho-Histone H3 was observed in cohorts with MYC on and exposure to either DDC or CCl_4_ (Figure 6C). Notably, the proliferation observed in MYC ON/DDC samples was diffuse, and not localized to the periportal areas of the liver lobule, further suggesting that these HCC's may not originate from the oval cells (Figure 6 and data not shown). Importantly, MYC induction alone had only a modest impact on hepatocyte proliferation within 17 days (Figure 6A–C). Thus, hepatotoxins can similarly promote continuous and diffuse hepatocyte proliferation irrespective of the targeted region of the liver lobule.

We previously reported that MYC is restrained from inducing the mitotic division of adult hepatocytes, and consequently for HCC formation, due to a p53-dependent cell cycle arrest [26]. To determine if loss of p53 is similarly necessary for MYC to induce HCC in the context of CCl_4_ or DDC, we examined p53 protein expression by Western analysis (Figure 6D). In contrast to our previous results, we found that adult tumor onset in MYC ON/CCl4 and MYC ON/DDC mice was not associated with a loss of p53 protein expression (Figure 6D). Thus, it appears that treatment with hepatotoxins allows for evasion of p53-cell cycle arrest upon induction of MYC overexpression, thereby allowing MYC to induce increased mitotic division and HCC. Interestingly, this is in agreement with our results demonstrating that p53 loss is not required for tumorigenicity in the neonatal liver [26].

### DDC and CCl_4_ treatment permit MYC-induced Cyclin B1 expression

MYC is thought to promote unrestrained proliferation and tumorigenesis in part by directly inducing the transcription of genes involved in cell cycle progression and mitosis [1], [33]–[35]. However, while MYC can rapidly induce mitotic entry in neonatal livers, oncogene activation initially results in hepatocyte hypertrophy in adult livers and only results in mitosis in this context upon neoplasia [26].

**Figure 7:**
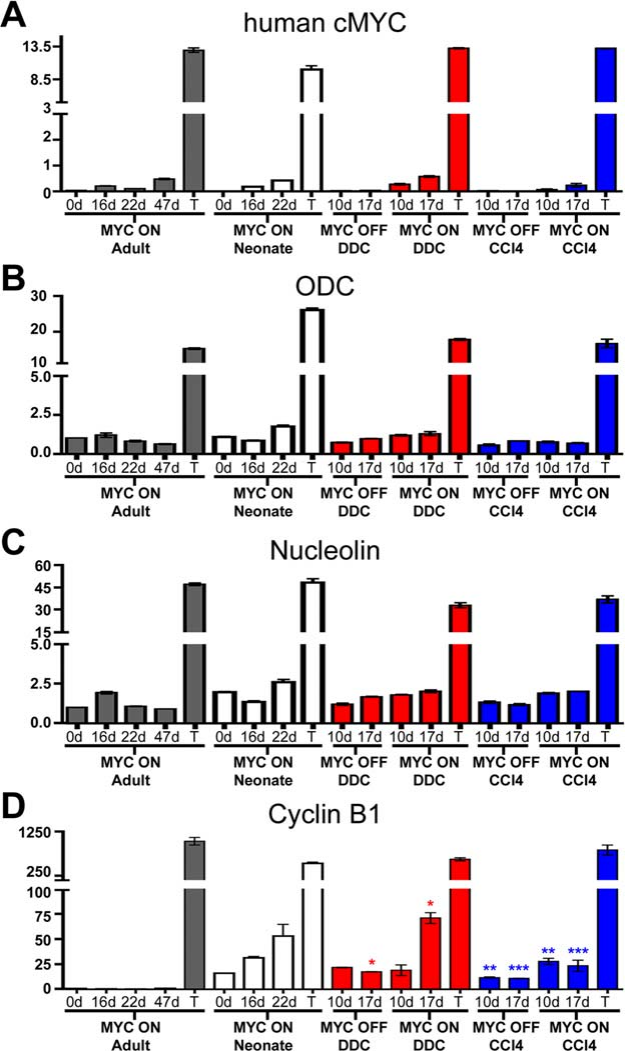
MYC-induced Proliferation and Tumorigenesis in Hepatotoxin-Treated Livers is Associated with Increased Cyclin B1 Expression. Real-time quantitative PCR of: (A) human c-MYC mRNA, (B) mouse Ornithine Decarboxylase (ODC) mRNA, (C) mouse Nucleolin mRNA, and (D) mouse Cyclin B1 mRNA. Statistical significance was measured using a Mann Whitney test (* P<0.05, ** P<0.05, *** P<0.05). Gray bar graphs represent normal adult livers in which MYC was overexpressed for the times indicated. White bar graphs represent normal neonatal livers in which MYC was overexpressed. Red bar graphs represent DDC-treated livers in which MYC was either overexpressed (MYC ON/DDC) or kept inactive (MYC OFF/DDC). Blue bar graphs represent CCl_4_-treated livers in which MYC was either overexpressed (MYC ON/ CCl_4_) or kept inactive (MYC OFF/ CCl_4_). mRNA was quantitated from four different liver samples per time-point.

To determine if the accelerated tumorigenesis in hepatotoxin-treated adult livers is due to differences in MYC's ability to induce the expression of genes involved in mitotic division, we measured the expression of known transcriptional targets of MYC by Quantitative Real-Time PCR (qRT-PCR). As a control, MYC expression was shown to increase in a temporal manner in all samples, with no significant difference observed in hepatotoxin-treated livers (Figure 7A). Similarly, no differences were observed in ODC and Nucleolin mRNA expression levels, two canonical targets of MYC's transcriptional activity (Figure 7B, C). However, when Cyclin B1 expression was examined, we discovered that MYC ON/DDC and MYC ON/CCl4 livers exhibited a 250 and 150 percent increase in Cyclin B1 mRNA, respectively, compared to MYC OFF/DDC and MYC OFF/CCl4 livers (Figure 7D). Cyclin B1 mRNA was also elevated in MYC OFF/DDC and MYC OFF/CCl4 livers relative to MYC ON and normal adult livers (Figure 7D). Interestingly, the pattern of Cyclin B1 expression in MYC ON/DDC and MYC ON/CCl4 is strikingly similar to the expression pattern in neonatal livers in which MYC expression was induced.

Our results suggest that when MYC is overexpressed in conjunction with DDC or CCl_4_ treatment, it may be more capable of transactivating genes involved in mitotic regulation in the adult liver. Moreover, the difference in Cyclin B1 expression could, in part, be responsible for the dramatic difference in MYC's ability to induce the mitotic division of hepatocytes in the context of DDC or CCl_4_.

## Discussion

Here, we show that two non-genotoxic hepatotoxins, DDC and CCl_4_, can cooperate with MYC to induce robust mitotic cellular division and the near immediate onset of liver tumorigenesis. Our results have implications for the mechanisms by which oncogenes such as MYC induce and are restrained from causing HCC. Previously, we have shown that MYC overexpression induces robust cellular proliferation in embryonic and neonatal hepatocytes, but, in marked contrast, induced cellular growth and DNA replication without mitotic cellular division in the adult liver [26]. We speculated that for MYC to induce liver cancer in adult hosts, the liver context must be changed, presumably through factors that stimulate hepatocyte proliferation. Indeed, both DDC and CCl_4_, toxins that target distinct regions of the liver [28]–[30] were found to markedly increase the ability of MYC to induce mitotic cellular division and permit the almost immediate onset of HCC. Our results suggest that oncogenes such as MYC can become latently active in the liver but only upon subsequent exposure to a hepatotoxin is their malignant potential manifested.

Our data are consistent with the general notion that the liver is most permissive to malignant transformation under circumstances when hepatocytes are undergoing cellular proliferation [26], [36]. Moreover, our observations further validate previous reports that MYC can cooperate with agents that modulate the liver and thereby stimulate hepatocyte proliferation. Most notably, MYC has been shown to cooperate with TGF-alpha and phenobarbital to induce accelerated tumorigenesis in the adult murine liver [37]. Our model recapitulates the latency of tumor formation associated with adult onset HCC. Hence, we should be able to define factors that either enhance or repress tumorigenesis. To our knowledge, our report is the first to describe that hepatotoxins cooperate with MYC overexpression to induce accelerated HCC specifically in the context of an adult murine liver.

A possible explanation for our findings is that MYC appears to be only capable of inducing the activation of Cyclin B1-Cyclin-dependent kinase 1 (Cdk1) in embryonic/neonatal hepatocytes or in adult livers treated with hepatotoxins. Elevated Cyclin B1 levels often precede the onset of tumor cell immortalization and aneuploidy [38], [39]. When we compared Cyclin B1 expression levels in the different cohorts of mice, we found that treatment with DDC or CCl_4_ was associated with elevated Cyclin B1 expression relative to a normal or MYC ON liver. Furthermore, when MYC was overexpressed in conjunction with either DDC or CCl_4_ we unveiled evidence for a further increase in Cyclin B1 expression levels. Notably, Cyclin B1 has been shown to be a direct transcriptional target of MYC [35], [41]. Hence, one likely explanation for these differences is that the Cyclin B1 promoter region may be more accessible to MYC in hepatotoxin-treated livers, possibly by regulation of chromatin structure [40]. Alternatively, the increased expression of Cyclin B1 could be attributed to mitotic signals from the regenerative response initiated in the liver by CCl_4_ or DDC damage. Future experiments will be directed at determining if MYC is directly activating the transcription of Cyclin B1 in hepatotoxin-treated livers.

Previously, we were able to show that a loss of p53 is associated with MYC-induced tumorigenesis in the normal adult liver [26]. In contrast, there was no evidence for p53 protein loss in MYC ON/DDC and MYC ON/CCl4 liver tumors, consistent with our observations in embryonic or neonatal MYC-induced tumors. Interestingly, Yin and colleagues recently showed that loss of p53 function cooperates with MYC to induce increased Cyclin B1 expression [41]. Our earlier report supports this mechanism in that loss of p53 in MYC-induced adult tumors was associated with elevated Cyclin B1 mRNA. Interestingly, we did not observe a correlation between p53 protein status and Cyclin B1 expression in hepatotoxin-treated livers. One possible explanation for this discordance is that, after exposure of the liver to DDC or CCl_4_, the already elevated levels of Cyclin B1 enable MYC to bypass the p53 checkpoint and further drive hepatocyte proliferation.

We hypothesized that one possible mechanism by which DDC facilitates MYC-induced HCC is by inducing oval cell expansion. Since these cells more closely resemble embryonic hepatoblasts, we posited that they may be more susceptible to MYC-induced tumorigenesis [42], [43]. However, when assayed by A6 immunohistochemistry, there was no evidence for a difference in oval cell number between MYC OFF/DDC and MYC ON/DDC livers. One possible explanation for a lack of change in oval cell number between MYC ON/DDC and MYC OFF/DDC livers is that some oval cells were undergoing neoplastic transformation, thereby losing their oval cell properties and were no longer expressing the A6 antigen. This idea is further supported by the fact that the first appearance of neoplastic foci and microcarcinomas in the MYC ON/DDC livers occurs as early as 10 days after MYC activation, and is predominantly located in the periportal area of the liver. Alternatively, these oval cells could have also lost their oval cell properties by quickly differentiating to immature hepatocytes. Our model should be useful in further investigating the role of oval cells in MYC-induced tumorigenesis.

In contrast to DDC, continuous exposure to CCl_4_ damages the liver by causing massive centrilobular necrosis and stimulating an inflammatory response through the activation of kupffer cells. Activated kupffer cells produce pro-inflammatory cytokines, such as Tumor necrosis factor-alpha (TNF-alpha), that can exacerbate chemical-induced liver injury as well as functioning as hepatic mitogens [44], [45]. TNF-alpha is commonly induced almost immediately after CCl_4_ exposure and is responsible for the activation of nuclear transcription factors that promote the expression of genes involved in cell cycle progression and mitotic division [44], [46]. It will be interesting to subsequently investigate the role of CCl_4_-induced TNF-alpha signaling in MYC-induced liver tumorigenesis.

In this report, we demonstrate that toxin-mediated liver damage is sufficient to accelerate MYC-induced HCC formation in adults. Interestingly, this effect is irrespective of the target of the hepatotoxin or whether it functions independently as a carcinogen. Our results are consistent with a model whereby oncogenic events, such as MYC overexpression, may be generally silent in adult somatic hepatocytes unless the host is exposed to a liver toxin that promotes a permissive environment for tumorigenesis.

## Materials and Methods

### Transgenic mice

The TRE-*MYC* transgenic line generated for these experiments was described previously [2]. The LAP-tTA transgenic line was kindly provided by H. Bujard [47]. MYC expression was activated by removing doxycycline treatment (100 µg/ml) from the drinking water of mice transgenic for both TRE-*MYC* and LAP-tTA.

### Tumorigenicity assays

MYC was activated in the liver by removing doxycycline treatment from the water. For toxin treatment, 6-week-old mice were either continuously fed a diet containing 0.1% DDC (Sigma-Aldrich, St. Louis, Missouri, United States) or injected intraperitoneally with carbon tetrachloride (Sigma-Aldrich) two times a week every week until sacrifice. CCl_4_ was diluted in mineral oil (Sigma-Aldrich) and administered at a dose of 1 µL per gram of mouse. Mice were monitored daily and were sacrificed when moribund with tumor burden. During necropsy, liver tissues were saved by snap freezing in liquid nitrogen or were prepared for histology by fixing in 10% buffered formalin for 24 h and then transferring to 70% ethanol until paraffin embedding. All procedures were approved by the Animal Care Committee at Stanford University.

### Immunohistochemistry

Tissue sections 4 µm thick were cut from paraffin-embedded blocks and placed on glass slides. Hematoxylin and eosin (H&E) staining was performed using standard procedures. The Stanford Histology Core laboratory prepared paraffin sections and performed H&E staining. A6 IHC was performed on liver tissue frozen in O.C.T. (−80°C), sectioned at 7 µm, and fixed in 100% acetone at −20°C for 10 minutes. Phospho-Histone H3 IHC was performed on formalin-fixed paraffin embedded tissue. Endogenous peroxidase activity was blocked in 0.3% H_2_O_2_ in methanol for 30 minutes, followed by blocking in rabbit or goat serum for 1 hour. The A6 rat monoclonal antibody was applied at a 1∶20 dilution for 1.5 hours at room temperature. The A6 antibody was a generous gift from Dr. Valentina Factor (National Cancer Institute, National Institutes of Health, Bethesda, Maryland, United States). The phospho-Histone H3 antibody (#9701, Cell Signaling Technology, Danvers, Massachusetts, United States) was applied at a 1∶100 dilution overnight at 4°C. Primary antibody incubation was followed by detection using either the rabbit anti-rat ABC Elite Kit or the goat anti-rabbit ABC Elite Kit (Vector Laboratories, Burlingame, California, United States) according to the manufacturer's protocol. Color was obtained using diaminobenzadine/nickel chloride (Vector Laboratories).

### Ki67 Immunofluorescence

Ki67 expression was examined by immunofluorescence using a mouse anti-human Ki67 monoclonal antibody (BD Biosciences, Palo Alto, California, United States) and the Vector M.O.M. Basic Kit (Vector Laboratories). Slides were deparaffinized in xylene and rehydrated in a graded series of ethanols, followed by antigen retrieval in a microwave for 14 min in antigen unmasking solution (Vector Laboratories, H-3300). Slides were then incubated in 100 mM glycine twice for 8 min to reduce fluorescent background. Slides were incubated in avidin for 10 min followed by biotin for 10 min using the Dako biotin blocking system (DAKOCytomation Corporation, Carpinteria, California, United States) and subsequently incubated for 1 h in M.O.M. IgG-blocking reagent diluted 1∶4 in PBS. Slides were then incubated for 1 h in mouse anti-human Ki67 monoclonal antibody diluted 1∶100 in M.O.M. diluent. Slides were washed in TBST for 3 times for 5 min to reduce background and were then treated with M.O.M. biotin-labeled anti-mouse IgG, diluted 1∶250 in M.O.M. diluent. Following another 3×5 min of TBST washes, slides were incubated for exactly 45 min in Cy3-conjugated streptavidin diluted 1∶800 in PBS (Amersham Biosciences, Piscataway, New Jersey, United States) in the dark. To visualize nuclei, slides were counterstained with 0.2 µg/ml DAPI. Ki67-positive cells were visualized by fluorescence microscopy.

### RNA Isolation and cDNA Preparation

Total cellular RNA was isolated from snap-frozen liver tissue using the RNeasy Kit (Qiagen, Valencia, California, United States) following the manufacturer's protocol. The amount of total RNA was quantified using spectrophotometric OD_260_ measurements. RNA was reverse transcribed using Oligo(dT)_12–18_ primers (Invitrogen, Carlsbad, California, United States) and SuperScript II Reverse Transcriptase (Invitrogen).

### Quantitative Real-Time PCR

qRT-PCR was performed on the ABI Prism 7700 Sequence Detection System (Applied Biosystems, Foster City, California, United States) using the SYBR Green PCR Master Mix (Applied Biosystems). PCR reactions were performed in triplicate in a final volume of 12 µL. The primer sequences were as follows: MYC forward 5′-ACCAGATCCCGGAGTTGGAA-3′, MYC reverse 5′-CGTCGTTTCCGCAACAAGTC-3′, ODC forward 5′-CTGTGCTTCTGCTAGGATCAATGT-3′, ODC reverse 5′-GCCTTAACACAAGCTAAACTTGCA-3′, Nucleolin forward 5′-GGAGGCCATGGAAGATGGAG-3′, Nucleolin reverse 5′-CACCTCTGCCTCCGAAACCT-3′, Cyclin B1 forward 5′-ACTTCCTCCGTAGAGCATC-3′, Cyclin B1 reverse 5′-GCAGAGTTGGTGTCCATTC-3′, Ubiquitin forward 5′-AGCCCAGTGTTACCACCAAG-3′, Ubiquitin reverse 5′-ACCCAAGAACAAGCACAAGG-3′. Thermal cycling conditions consisted of the following steps: 95°C for 10 minutes, followed by 40 cycles of 95°C for 15 seconds, 57°C for 30 seconds, 72°C for 30 seconds, and a dissociation stage consisting of 95°C for 15 seconds, 60°C for 15 seconds, and 95°C for 15 seconds. MYC, ODC, Nucleolin, and Cyclin B1 mRNA expression levels were normalized to Ubiquitin expression levels and expressed relative to mRNA levels in a normal liver.

#### Western Blot

Western analysis was performed using conventional techniques. Liver tissues were disrupted and protein was isolated using a tissue homogenizer in NP-40 lysis buffer. Proteins were electrophoresed on 7.5% Tris-HCl polyacrylamide gels and transferred onto PVDF membranes. The membrane was blocked in 5% nonfat dry milk solution in TBST at 4°C overnight. p53 protein expression was detected using the NCL-p53-CM5p rabbit polyclonal antibody (Novacastra, VisionBiosystems, Inc., Norwell, Massachusetts, United States) at a 1∶500 dilution. As a positive control, we used liver tissue from mice injected with a p53 expressing vector.
